# ‘Making little ethical decisions all the time’: examining an ethical framework for consumer and community involvement in research, a co-produced ethnographic study

**DOI:** 10.1186/s12910-025-01355-6

**Published:** 2025-12-22

**Authors:** Ruth Cox, Matthew Molineux, Melissa Kendall, Elizabeth Miller, Bernadette Tanner

**Affiliations:** 1 Occupational Therapy Department, Queen Elizabeth II Jubilee Hospital, Coopers Plains, QLD Australia; 2 Discipline of Occupational Therapy, School of Health Sciences and Social Work, Griffith University, Gold Coast, QLD Australia; 3 Acquired Brain Injury Outreach Service and Transitional Rehabilitation Program, Princess Alexandra Hospital, Buranda, QLD Australia; 4 School of Health Sciences and Social Work, Griffith University, Gold Coast, QLD Australia; 5 Consumer Co-Researcher C/O Occupational Therapy Department, Queen Elizabeth II Jubilee Hospital, Coopers Plains, QLD Australia

**Keywords:** Consumer and community involvement, Patient and public involvement, Patient engagement, Patient partnership, Ethical considerations, Healthcare research, Biomedical research, Co-production

## Abstract

**Background:**

Consumer and community involvement (CCI) is widely recognised as an ethical imperative in health and biomedical research. However, there is a lack of evidence and guidance regarding ethical approaches. The aim of this research was to test and refine an existing ethical framework for consumer partnerships in research to enhance understanding of ethical issues and approaches to CCI in research.

**Methods:**

A sub-analysis of a co-produced ethnographic study which explored the processes and outcomes of consumer engagement over three and a half years in a PhD research partnership, was conducted against an existing ethical framework for CCI. The framework included organisational ethics, research integrity, relational ethics, and research ethics. Participants included four consumers, two academics, and a PhD candidate in an Australian capital city. Two consumer co-researchers collaborated in this study across the research cycle. Data were obtained over three and a half years from six interviews, six focus groups, monthly online logs, field notes, and a reflexive diary. Descriptive statistics and qualitative content analysis were used to analyse 2035 units of data.

**Findings:**

A total of 1911 (93.9%) units of data aligned to an ethical category. Hence, the research team were constantly encountering ethical decision-making. A combination of organisational ethics and relational ethics was most frequently coded (30.1%, *n* = 576), followed by relational ethics alone (24.3%, *n* = 465), and research integrity and relational ethics (*n* = 229, 12.0%). Qualitative analysis identified some ethical tensions and many more practical and planned ethical approaches to support meaningful research partnership and positive research processes and outcomes. Examples and quotes are provided against each of the four ethical categories to illustrate and expand on the framework. An update to the framework is provided.

**Conclusions:**

The updated framework highlighted the complexities of CCI and focused beyond traditional research ethics to include relationships, organisational factors, and research integrity. The narrative of ethical issues being a challenge to overcome in CCI, needs to change. An emphasis on adopting a proactive approach to promote ethical and authentic team power sharing, reflection, and active communication is needed.

**Supplementary Information:**

The online version contains supplementary material available at 10.1186/s12910-025-01355-6.

## Background

Consumer and community involvement (CCI), also termed patient and public involvement, consumer engagement, co-production, and patient and family engagement, is widely recognised as an ethical imperative in healthcare and biomedical research [1–4]. A detailed analysis of terminology is beyond the scope of this paper, but is important to recognise that the array of terms may be interpreted differently across disciplinary, jurisdictional, and international contexts [4, 5]. CCI nomenclature is used here for consistency with guidance from Australia’s peak body for the support of health and medical research [6]. The origins of the term ‘consumer’ in Australia possibly stem from the 1970’s consumerist movement when ‘patients’ with lived experience of mental illness wanted more control over medical decisions about their own bodies, health funding, and health policy [7]. It is recognised that ‘consumer’ may imply choice and power which is frequently not the case in research [8]. The focus of this paper is on collaborating with consumers in research on an individual level as part of a research team which included academics and service providers, rather than at a community level. Other participatory approaches such as Community Based Participatory Research [9, 10] and Citizen Science [1, 11] aim to harness collective public knowledge to address complex local challenges. They have a more democratised community-led approach [12] than this study. However, participatory research approaches share many underlying philosophies and face similar challenges of tokenism and social exclusion [13]. Hence the literature review for this study encompassed a range of participatory designs.

Ethical approaches to CCI are essential and the lack of evidence-based guidance has been recognised [2, 11, 14]. Ethical conduct is more than just doing the right thing, it involves acting in the right spirit out of enduring respect and concern for others [15]. Authenticity is core to ethical partnerships as it encompasses a genuine and transparent commitment to valuing CCI, and to treating consumers with respect [16]. One size does not fit all for CCI in research [6, 17, 18]. For the purposes of this paper, an authentic approach to CCI is characterised by consumers and health professional researchers working together iteratively over the lifespan of the study to jointly steer the direction, decisions, and implications for practice and future research [4, 19]. Ethical issues that may be encountered in CCI include consumer partner confidentiality, anonymity, consent, emotional support, reward, recognition, and reimbursement [2, 20, 21].

In a sub-analysis of a systematic review, Ludwig et al. (2021) used Beauchamp and Childress’ Principles of Biomedical Ethics (autonomy, non-maleficence, beneficence, justice) to examine ethical considerations for engaging frail and seriously ill consumer partners in research. In autonomy, promoting the desired level of involvement, addressing relational and intellectual power, and facilitating research knowledge were highlighted. Non-maleficence encompassed protection from financial burden, and physical and emotional suffering. For beneficence, helping others, demonstrating value-add, and supporting patient partners were identified. Justice included appropriate representation, mutual respect, and balancing risks and benefits. A qualitative study examined patient partners’ experiences in an arthritis research advisory group [22]. Consumers emphasised that being heard, mutual respect, equal relationships, co-building social relationships beyond research-focussed interactions, and minimising risks of physical and emotional impacts were essential for building ethically sound CCI practice [22]. Another qualitative investigation focussed on early-career researchers’ views of ethical dimensions of patient engagement in research [23]. The issues most frequently raised, in rank order, included professionalised patients, consumer remuneration, reward and recognition, potential misuse of grant funds gained from CCI, power sharing, confidentiality, exploitation of vulnerable people, paternalism, and educating patients about research and research integrity [23].

The above literature focused on the perspective of only one stakeholder group (i.e. consumer or health professional researchers only) which is a limitation given the importance of consumer and health professional researchers working together to identify ethical approaches to partnered research [1, 2, 21]. This issue was addressed in a study where community members, ethicists, and academic researchers established a stakeholder-driven ethical framework for CCI through several consensus rounds [24]. The framework outlined a core vision of just and equitable research with a collective ethos and consideration of individual and group perspectives, translation for improved health, capacity building, and power sharing [24]. Additionally, community informed risks and benefits analysis, relational dynamics, and emphasising accountability were incorporated [24]. A qualitative study examined ethical considerations from the perspectives of health professional researchers, patients, and families in end-of-life and palliative care research [16]. The most frequently described ethical challenges were minimising engagement burdens, dealing with death and illness, and paternalism or gatekeeping where researchers restricted CCI opportunities due to concern for consumer well-being. Ethical issues went beyond the classical principles of biomedical ethics to include relational ethics [16]. In another study, case studies were used to examine ethics in citizen science [1]. It was concluded that ethical considerations in partnered research go beyond traditional biomedical and health research ethics. Relational and moral complexities of collaboration, sharing power, and democratic decision-making were incorporated into a model with seven features: framing work, role work, emotion work, identity work, reason work, relationship work, and performance work [1].

In a scoping review, Martineau et al. (2020) reported the most frequently cited ethical themes from literature discussing CCI in research. The top five issues were lack of resources, challenges in consumer partner selection, lack of support, absence of a shared vision, and tokenism. Similar to others [1, 16], the literature and stakeholder consultation highlighted that the discourse regarding partnered research needs to extend beyond a research ethics framework and protecting consumers from harm. Four ethical frameworks for patient partnerships in research were presented in a model that included organisational ethics, research integrity, relational ethics, and research ethics [2].

The research team for this current paper, included two academics, a PhD candidate, and two consumers. They collaborated over three and a half years on six studies for a PhD. During the partnership they became increasingly aware of the importance of expanding knowledge and evidence regarding ethical approaches to authentic CCI which concurred with other authors [23, 25, 26]. The literature summarised above was examined to identify an ethical framework in CCI against which to review the team’s experiences. During three two-hour workshops it was agreed that the Martineau et al. (2020) framework was most applicable. The strengths of this framework were that it included both consumer and health professional researcher perspectives, was developed from the literature, and included consultation with a stakeholder committee inclusive of consumers, health professional researchers, research funders, and human research ethics committee members. Its visual appeal and plain language were also positive aspects. The aim of this paper is to enhance understanding of ethical issues and approaches for CCI in research by examining data drawn from a co-produced ethnographic study of a three and a half year research partnership in a PhD in order to test and refine the Martineau et al. framework [2].

## Methods

### Research objective

The research objective was to test and refine an existing ethical framework for consumer partnerships in research to enhance understanding of ethical issues and approaches to CCI in research.

### Design

A sub-analysis of a co-produced ethnographic study was conducted. The main study explored the processes and outcomes of consumer engagement over three years in a PhD research partnership. The main study is reported elsewhere and included discussion of optimal approaches, impacts, and benefits and challenges of consumer partnerships in a PhD from the perspective of the consumers, academic supervisors, and the student [14]. Ethnography uses multiple data collection methods to explore how a cultural group interacts and the meaning of the interactions [27]. Given the examination of values, purpose, and power are at the core of ethnography [28] it was a highly suitable design for this study. The Consolidated criteria for reporting qualitative research (COREQ) checklist guided reporting of this study [29].

### Setting

The study was conducted in a state capital city hospital and health service and a university in Australia.

### Participants and recruitment

Expressions of interest were sought via email from approximately 125 consumer partners and volunteers of a large Australian metropolitan hospital and health service. The email encouraged interested consumers to discuss the ethnographic study and CCI role expectations with the PhD candidate. Interested consumers were then invited to participate in a relaxed and supportive interview with the PhD candidate and at least one academic supervisor. Three consumers were recruited. They were all female. The process was repeated at month five as one consumer withdrew due to self-reported time limitations. An additional consumer (male) was recruited and withdrew in month eight due to personal circumstances. The remaining two consumers were partners in the research team for three and a half years. The PhD candidate (female) and two academic supervisors (one male, one female) were not formally recruited but provided informed consent.

### Consumer and community involvement

The consumer co-researchers collaborated in six studies within the PhD and this sub-analysis of the main ethnographic study across the research cycle. Consumer collaboration occurred during 41 meetings across three and a half years with many additional interactions between meetings. Consumers were integral to study decision making including data interpretation and co-authorship of a scoping review [30], design, conduct, and publication of a second scoping review [31], an e-Delphi study [32], an evaluation of a short term research advisory group [33], a learning and development needs analysis [34], and the main ethnographic study [14]. Additional File 1 includes the title, aims, and CCI strategies across the research cycle for the six PhD studies. Team achievements included co-authorship of six publications, four national and two international conference oral presentations, conference posters, many local presentations, social media posts, and online consumer facing newsletters. Additional CCI during the PhD included a six month research advisory group [33] and inclusion of an additional consumer and an existing health service consumer partnerships committee for data interpretation of another study [34]. Two microgrants assisted in remunerating the two long-term consumers to a total of AUD$750 each. When the term research team is used in this paper, it includes the two long-term consumer co-researchers.

### Data collection

Data were collected from December 2019 to December 2022 through interviews, focus groups, monthly online logs, field notes and a reflexive diary for the overarching ethnographic study [14]. More detail is provided in Table 1. The interview and focus group guide incorporated discussion topics about what went well and what did not go well in the research partnership during the previous six months; degree of consumer influence; benefits and challenges; ethical issues; and learning and support. The research team reviewed the guide before each interview or focus group to enhance their preparation and to ensure that it was still relevant and comprehensive. The guide for the final focus group is included in Additional File 2. Focus groups were used after the six-month interviews as team members valued the opportunity to hear others’ reflections and to compare with their own experiences when responding to the guide. The monthly online log included individual reflections on satisfaction, difficulty, benefits, and challenges of CCI during the PhD. The focus of this ethnographic study was on the core research team. Hence data collected directly from the research advisory group and consumer partnerships committee participants were not analysed here. The research team’s (including consumers’) perspectives regarding interactions and outcomes of the collaboration were included.

**Table 1:** Data collection strategies

Data source	Participants	Collection strategy	Details
Field Notes (FN)	4 consumers 2 academics PhD candidate	PhD candidate took field notes; academics reviewed at supervision	107 pages from 41 meetings and 5 focus groups over 36 months
Interviews (IV)	4 consumers 2 academics	PhD candidate interviewed participants individually face to face for 45 min–1 h	6 interviews; at 6 months; audio recorded; hospital staff office setting
Focus Groups (FG)	2 consumers 2 academics PhD candidate	External researcher with high level capabilities in qualitative research and consumer engagement facilitated face to face (except the last focus group where one consumer participant was online) for 90 min each	5 focus groups; 6 monthly from 12 to 36 months; audio recorded; community rehabilitation service meeting room except final focus group at university meeting room
Online reflection log (Log)	4 consumers 2 academics PhD candidate	Monthly anonymous online; log entries coded as consumer or non-consumer	Monthly for 36 months
Reflexive diary (RD)	PhD candidate	Recorded on an ongoing basis	As required for 36 months

### Data analysis

Deductive qualitative content analysis [35] was used. A deductive approach was suitable as the study objective was to test an existing ethical framework for CCI in research [2] against data collected [36]. The first author developed category definitions and codes based on the relevant publication [2]. These categories and codes were reviewed by the research team and minor adjustments made. NVivo software was initially used to code data [37]. Data were then downloaded to a spreadsheet which included data source, code, and original text as this format was easier for the consumer co-researchers to access. A total of 2035 units of data were available for coding. For the purposes of this study a unit of data was considered to be a single concept consisting of one to approximately five sentences. The first author conducted line by line coding of ethical issues on approximately 6% of the data (114 units) using a spreadsheet with the four categories in separate columns and a drop-down list for each code. The research team met to review the coding and agreed to add coding of positive examples of ethical approaches in the CCI partnerships. The final categories and codes are included in Table 2. The first author coded 26.3% of data (*n* = 536) including recoding the previously coded 114 units. This was checked and approved by the team and the remainder of data coded. Results were presented to the team with 80 examples of indicative quotes against the categories. The team reviewed draft findings during a 1.5-hour meeting and minimal changes were made. At that meeting, the team decided that quantification of the number of units of code per category would be useful to demonstrate relative importance to day-to-day research team functioning. The first author kept a reflexive diary, memoed during data analysis, and maintained a log of research team decisions to further enhance trustworthiness.

**Table 2:** Coding guide based on Martineau et al. (2020) ethical frameworks for patient partnership in research

Ethical framework category definition	Examples from Martineau et al. (2020) used to guide coding
**Organisational Ethics**: Practices, programs, structures, rewards and leadership used by managers and employees to encourage ethical and responsible conduct, and to facilitate ethical and responsible relationships with stakeholders. Includes ethical decision-making and behaviour from organisational actors, and management of misconducts.	• Organisational support • Evidence based policies • Training regarding principles and methods • Researcher knowledge of principles and process for meaningful CCI • Reward and recognition • Resources for researcher time and costs • Effective organisational communication • Equity of consumer selection and access to CCI opportunities including consideration of: - unstable health conditions - diversity and hard to reach populations - representativeness of consumer experiences compared to larger targeted population • Pressure on individual consumers to engage • Reimbursement of costs to consumer partners • Compensation and remuneration of consumer partners including: - Fair renumeration - Impacts of financial reward on motivations - Effect on individuals’ government benefits • Logistical and practical enablers and barriers including time required • Consumer partner wholistic health – mental, social, physical • Evidence base and evaluation framework to guide and demonstrate value and impact • Research culture
**Research Integrity**: Concerned with the way scientific research is implemented including scientific rigour, honesty, reliability, referencing and plagiarism, authorship, conflicts of interest management, and grant application and fund management. The responsibility of researchers and research professionals and research institutions and guided by national and organisational policies and guidelines.	• Consumer knowledge about research methodologies and scientific methods • Consumer conflicts of interest - Dual healthcare worker or service provider role - Relationship with industry groups - Consumer advocate, or member of a patient advocacy organisation • Researcher conflicts of interest - Dual role as researcher and healthcare provider • Co-Authorship - Medical journals reluctance to publish consumer co-authored research - Timelines and budget for preparation of manuscripts • Emphasis on certain research - Prevalence disease and availability of consumers - Obtaining results that can rapidly be translated to care - Study rigour or trustworthiness - Grant applications - Funding management
**Relational Ethics** Focuses on relationships and connections between individuals, and highlights ethical values and concepts such as mutual respect, engagement, and collaborative environment. Includes a shared vision and the quality of research team relationships with consumer partners including the meaningfulness of consumer partnerships and influence on research processes.	• Researcher trust in value of consumers’ contribution • Consumer research knowledge and expertise • Tokenistic consumer engagement • Consumer exclusion from specific research phases, particularly dissemination and communication • Shared vision - Understanding roles - Consumer objectivity - Benefits and challenges of consumer engagement • Consumer partner turnover • Agreement and conflict - Research priorities - Research result dissemination activities and tools - Generalisable outcomes versus highlighting results leading to improved services and/or public policies • Power differential that constrains consumer contribution and/or leads to conflicts • Burden and benefits to consumer partners • Efficiency and dynamics of meetings
**Research Ethics** Encompasses ethical involvement of consumers as research participants including respect, autonomy, concern for welfare, protection from risks, and justice. These ethical principles are included in national and institutional guidelines and are implemented by Human Research Ethics Committees (HREC).	• Data confidentiality • Incidental findings about patient participants • Participant recruitment • HREC application • Impacts on study participants • Conforming to ethically cleared protocol

### Positionality

The qualitative content analysis included interpretation of latent content given data encompassed opinions, attitudes, perceptions and experiences [36]. Hence, it was critical to reflect on the positionality of the research team including values, life experiences, and philosophies relevant to the study [38]. All research team members had a strong commitment to ethical and authentic CCI. Ethical issues were a standing agenda item at all 41 team meetings. Three team members were health professionals (one hospital and community occupational therapy director, one occupational therapy academic, and one psychology-trained community rehabilitation researcher). The two long-term consumers were both retired. One had worked as a pathology scientist and one as a primary care policy advisor with an organisational psychology qualification. One consumer had more than seven years as a consumer partner and the other was new to CCI. The diversity of team lived experience included a disabling autoimmune condition, LGBTIQ + identity, sibling of a person with a severe acquired brain injury, primary carer of an older person with advanced Parkinson’s disease, family member of elderly parents or relatives with a history of multiple hospitalisations, and close friend of a person with long-term mental health issues.

## Findings

### Updated ethical framework for CCI in research

**Fig. 1:**
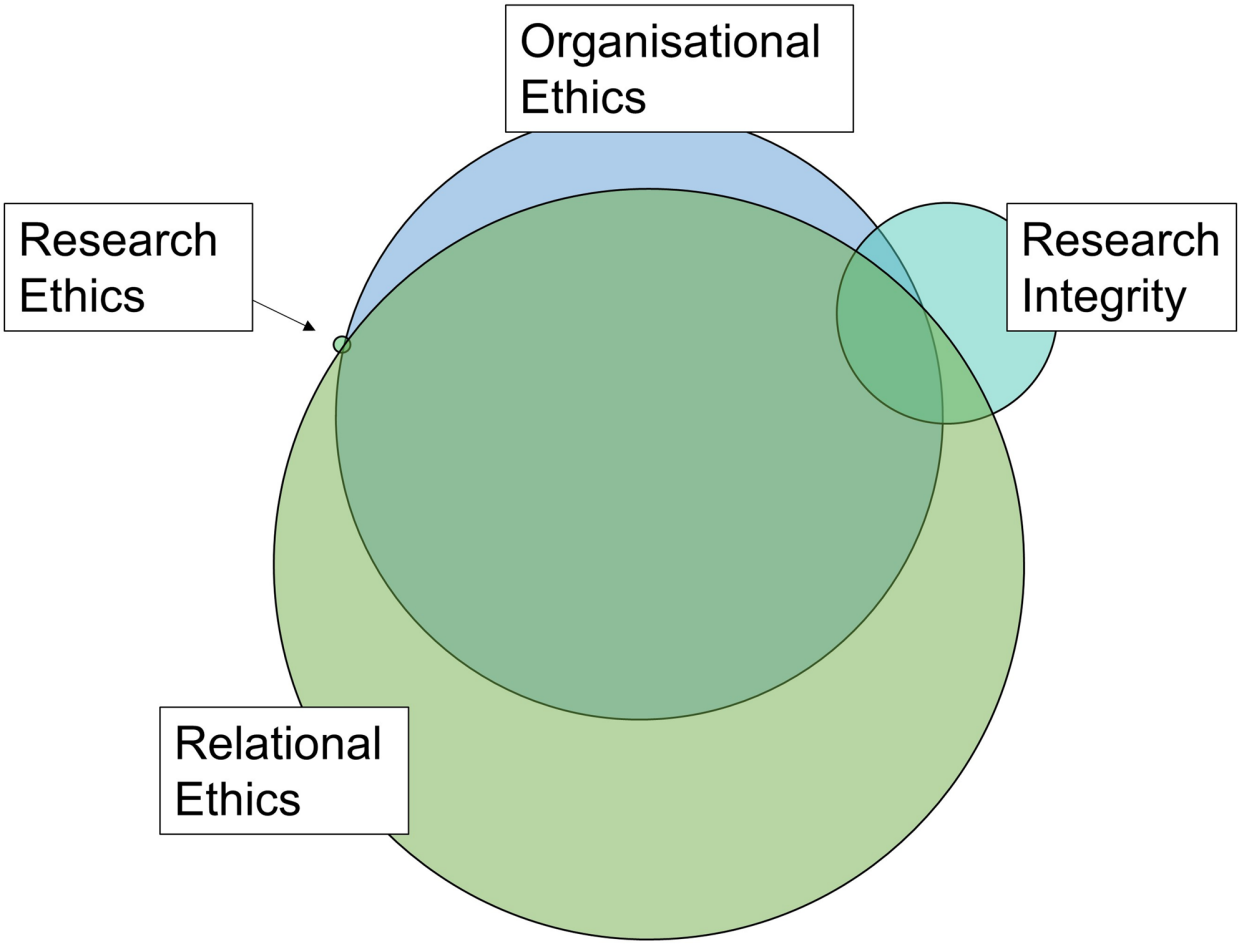
Updated Ethical framework for consumer and community engagement in research

Figure 1 provides an update to the Martineau et al. model (2020) based on the quantitative and qualitative data. It highlights the substantial overlap of the ethical categories and depicts the higher frequency that relational ethics and organisational ethics were encountered during the study. The sections below expand on the findings which led to the updated framework.

### Quantitative data

There were 2035 units of data to analyse. Less than 7% (6.1%, *n* = 124) of the units did not align with any ethical category. Of the units that were coded to an ethical category (*n* = 1911), 54.4% (*n* = 984) were coded to more than one. A combination of organisational ethics and relational ethics were most frequently coded (30.1%, *n* = 576), followed by relational ethics alone (24.3%, *n* = 465), organisational ethics alone (19.4%, *n* = 370), and research integrity and relational ethics (*n* = 229, 12.0%). When three categories were coded (4.4%, *n* = 85) this was usually a combination of relational ethics, organisational ethics, and research integrity. Approximately 4% of data (*n* = 82, 4.3%) were coded as research integrity alone, 3.2% (*n* = 62) as organisational ethics and research integrity, and the rest of the combinations, including research ethics alone, were coded less than 1% of the time.

### Qualitative data

Given 93.9% of the units of data (*n* = 1911) included content coded to an ethical category, it was clear that the research team were constantly engaging in ethical decision-making. Sam, an academic, highlighted in the final focus group (FG-36mths) that the team implemented, *“…ethics in practice as opposed to formal ethics … particularly when we’re talking about consumer engagement ‘cause you’re making little ethical decisions all the time….”* The qualitative analysis identified some ethical tensions and many more practical and planned ethical approaches which resulted in a meaningful research partnership and positive research processes and outcomes. The description below uses the data to illustrate and expand on the four ethical categories of Martineau et al.’s (2020) framework. Where data were coded across more than one category, this is indicated. The data source is shown using the format pseudonym- participant category-data collection method-timing e.g. -Chris-Consumer-IV-6mths.

### Relational ethics

Building and navigating authentic and productive relationships within the team was a focus of much data. The academics and PhD candidate demonstrated that they greatly valued the consumer contributions and trusted their insights. A consumer commented, *“… I think that openness and the flexibility of it*,* of the team*,* of [academic names] has been really*,* really*,* nice and I think affirming that you know our contribution is valued and taken on board.”*-Alex-Consumer-IV-6mths. Team members worked together to build relationships as illustrated by this quote, *“I felt this equal partnership when discussing [new consumer’s] orientation. The goodwill in terms of making [them] part of the team was also expressed several times.”*-FN-Mtg6-6mths. From early on, an openness by all parties to listen and discuss was evident and was an enjoyable aspect of the process. Ashley remarked, *“I always like to share my ideas and then see what other people’s thoughts are on that. That was what I really*,* really enjoyed in that group environment.”*-Ashley-Consumer-IV-6mths.

It was acknowledged that partnering with consumers may have reduced efficiency of meetings but that this was outweighed by the benefits. Sam remarked, *“… I think what it adds is a richness that you would not get if it was the three of us [non-consumers…. There’s a balancing act*,* and so I think the slowing down is not a bad thing at all*,* because you get so much more out of this whole group doing stuff.”*-Sam-Academic-FG-12mths. Over time, capability was built which led to enhanced efficiency. A consumer described this experience, “*Something’s changed…. at the beginning there was a lot of reading*,* a lot of papers*,* we had to discuss a lot of things.… now … we’ve gotten into a bit of a groove*,* and we whiz through it a little bit faster because you’re not having to stop … as much to explain things.”*-Alex-Consumer-FG-18mths.

Learning and development to overcome any lack of consumer research knowledge so as to enable meaningful partnerships was evident. This was not an easy juggle and the PhD candidate reflected on not wanting to distress or overburden the consumer co-researchers but that new learning was a shared phenomenon.-FN-Mtg10-9mths. Furthermore, the substantial time and resources to provide learning and development led to consumer empowerment, “*My knowledge has increased dramatically and hence … I have felt more comfortable in speaking…. I’ve noticed that … subtle change in [my] demeanour and [my] participation*,* [I] clearly feel more empowered…”*-Chris-Consumer-FG-36mths. This was an illustration of the overlap between relational and organisational ethics.

There were times when the motivation of consumers was potentially not aligned with research aims and this required a proactive approach. For example, when an external consumer contributed to data analysis it was noted that, *“[external consumer name] very much was there to advocate for improved learning and development of consumers. He was able to bring some useful insights to the research when pointed in the right direction.”-*FN-Mtg40-36mths. There were also occasions where a consumer’s response or reaction challenged the PhD candidate emotionally.

*“… I found her [consumer] to be very negative about [health service name] staff. …people do care and they also have very busy and often stressful clinical responsibilities which may sometimes limit their ability to engage consumers. I felt the need to say that I did not agree with her and to point out that we were getting off track. I hope I didn’t embarrass her or anyone else. I was quite surprised by how emotional my response was and still is a day later.”*-FN-Mtg9-8mths.

The strength of the collegial relationships and the importance of embracing disagreements was raised, *“… usually it’s dealt with rather than just … let’s move onto the next topic and pretend it didn’t happen.”*-Regi-PhD_Candidate-FG-12mths.

### Organisational ethics

The time, planning and resources required for health professionals to meaningfully collaborate with consumers were raised throughout the three and a half year study. An academic noted that time spent in consumer partnership meetings was *“… essentially time I would devote to another PhD student.”*-Sam-Academic-IV-6mths. Also, *“I found the juggle of so many consumer engagement activities across the research cycle and now … the RAG [research advisory group] quite challenging as there was a lot to consider to tailor the work to so many different people and tasks.”*-Non-Consumer-Log-24mths. Supporting accessible parking, venues, and meetings for a consumer with mobility, pain, and fatigue issues was essential when planning a co-presentation (FN-Mtg23-20mths), arranging face to face (FN-Mtg3-4mths) and online meetings (Alex-Consumer-FG-18mths), and when conducting focus groups (FN-Mtg28-24mths).

Organisational considerations of consumer burden coupled with impacts on relationships and group functioning were described. Incorporating a longer break period over December-January was factored in to accommodate consumer fatigue despite it being a holiday period and potentially productive time for the PhD candidate.-FN-Mtg16-13mths. Monthly log entries by consumer and health professional researchers often noted that the partnership was challenging but *“… enjoyable and rewarding.”*-Consumer-and-Non-Consumer-Log-12mths. Ensuring that consumers felt empowered to say ‘no’ was important: *“… it’s good that people can be open and honest and say*,* you know*,* pass. I’m at the beach. Someone else can do it.”*-RegiPhD_Candidate-FG-36mths.

The diversity gaps within the team (including no cultural diversity, no First Nations member, and high education levels) were discussed at all focus groups. Involvement of a research advisory group, and consultation with an existing health service committee for two studies addressed some of these gaps. However, the unresolved organisational and relational ethics challenge of how to effectively and ethically collaborate, rather than consult, with consumers who had lower levels of health literacy was not easily resolved. An academic was concerned regarding tokenism and the wish to be, *“… engaging with people in a meaningful*,* authentic way…. rather than having the token person from whatever background who maybe isn’t able to read all of the information and contribute meaningfully to the group and maybe become distressed because of it.”*-Sam-Academic-IV-6mths.

The perception that experienced consumers became professionalised and less able to present a fresh perspective was not the experience of the team. This concept was coded against organisational ethics, research integrity and relational ethics, and was seen as beneficial, *“The advantage I see is that more experienced consumers such as [consumer name] bring the ability to link their learning with the work at hand and build on it. They also provide advanced advocacy skills and the ability to bring stakeholders together.”*-FN-Mtg9-8mths.

University processes for PhD candidate assessment were identified as not being inclusive of consumer co-researchers and this was coded against organisation and relational ethics:

*“[I] flagged the potential date for final PhD seminar but I made it clear that this is not negotiable as [academic name] needs to coordinate multiple people to be available from a uni marking perspective. [Consumer name] noted that the date didn’t suit her but also did not attempt to negotiate a change which felt weird to me but a relief. It is one area where the consumers have no influence*,* which is why it felt weird. My discomfort was an indicator to me that it is business as usual for us all to have our preferences considered which is part of power sharing. When it doesn’t happen*,* it feels wrong.”-*FN-Mtg39-35mths.

Conversely, a consumer remarked that attending university milestone seminars and reading the confirmation documents was a positive experience as it, *“…helped me to see where we’d come from*,* where we were going*,* where we’d been.”*-Chris-Consumer-FG-12mths.

There were challenges due to a lack of organisational procedures for consumer remuneration despite having a small grant, *“…the money is in my research cost centre*,* but getting it out to give to [consumer names] (laughs) is a different thing altogether. So*,* I thought I had it all sorted and then they’ve [the organisation] gone radio silence on me so I’m a bit worried*,* which I find intensely frustrating.”*-Regi-PhD_Candidate-FG-18mths. A commitment to positively influencing traditional research culture was evident. An academic discussed lobbying and raising awareness about CCI at the university, *“I’ve asked [PhD Candidate name] to do a seminar to the School…. we’ve got these great examples of outcomes and processes and hints and tips … to influence other students*,* but also hopefully the system….”*-Sam-Academic-FG-36mths. Members of the team co-designed introduction to CCI videos with other members of the health service to, *“… help to equip staff and consumers for research partnerships…”*-Non-Consumer-Log-18mths. The stronger CCI research culture at the health service compared to the university was acknowledged. The PhD candidate relied on health service organisational systems and processes to keep the momentum going.-Regi-PhD_Candidate-FG-36mths.

### Research integrity

The substantial consumer contribution to challenging assumptions and adding new perspectives in the research planning phase was evident: “*It’s making the process visible…. It’s sort of opening it up … often those things go on in the background and you don’t really take much notice*,* but by stopping and talking about it … it does bring it out into the open and it makes you think more….”-*Sam-Academic-FG-36mths. This quote was coded as both research integrity and relationship ethics.

During the study execution phase the consumers assisted with recruitment and data analysis which was highly advantageous. There were times when the PhD Candidate felt she had to negotiate with a consumer who wanted faster action, *“There have been several emails back and forward with [consumer name] about the recruitment and I have been putting the brakes on this as I want this to be a well thought through process that is not rushed*.”-FN-Mtg14-11mths. Additionally, collecting data that was representative of all experiences could be challenging, “*It seems that people screen out the negative and focus on the positive which of course maintains momentum and goodwill but if we want to learn from this we do need to remember about the negatives.*”-FN-FG-12mths.

When discussing research dissemination and translation, the impact of collaborating with consumers was raised, *“… I think it is a massive influence from having [consumer names] involved because it’s just not good enough just to publish it*,* because I’d feel terrible if they’ve done all this work and then just a few people read it….”*-Regi-PhD_Candidate-FG-36mths.

The value of team work in genuinely collaborating in this phase was noted:

*“… the team pulled together really well to give me answers/opinions/suggestions regarding … the eDelphi manuscript. A lot of discussion was generated*,* and people seemed comfortable to provide their views. Not everyone agreed and we worked through pros and cons to reach a conclusion…. so that I could continue with the next draft.”*-FN-Mtg29-25mths.

Co-authorship and co-presentations provided much satisfaction but also some anxiety, *“The hardest part was being nervous about and wondering how the Showcase would be received!”*-Consumer-Log-12mths. Getting the right balance between conference presentations and preparation of peer reviewed publications required negotiation as the consumers were eager to share results quickly and locally in order to influence changes in systems and practice.-FN-Mtg18-16mths. This was also coded against relationship ethics.

Health professional researcher conflicts of interest were evident. For example, paternalism when working with a consumer co-researcher was met with ‘gentle assertion’:

*“[Consumer name] had a two-week long hospitalisation. We talked about her nerve pain and then her weakness and balance… She then gently pushed back when [non-consumer names] went into health professional and advice mode asking her about falls prevention … then changed the subject…. [Consumer name] handled this situation well and made it clear where her boundary was regarding our role and her health situation.”*-FN-Mtg39-35mths.

Keeping the boundaries clear between being a research colleague and health professional were important, *“I have also been asked for health advice on several occasions by [consumer name] which I am also happy to provide…. I make suggestions for accessing services*,* but I do not offer to provide services or to give ‘special’ access.”*-FN-Mtg12-10mths.

The proactive approach to ethical considerations including confidentiality of research findings assisted decision-making for a consumer who was contacted by an external researcher to share her CCI experiences, *“… because we constantly talk about ethics and it just didn’t sit right with me … it would be very stressful for letting something slip or whatever*,* so I thought it would be better not to even enter that territory.”*-Alex-Consumer- FG-18mths. Providing detailed choices and information to consumers regarding their privacy when posting on social media, in conference presentations, and in publications was key to research integrity and relational ethics. For example, *“I’m not on Facebook or Twitter*,* but I thought*,* who’s going to be knowing about me that I don’t know?*”-Chris-Consumer-FG-12mths. Maintaining privacy when documenting positionality for publications required discussion so that informed choices could be made about what team members wanted to reveal about themselves.-FN-Mtg30-26mths. This situation was coded against research integrity, relational ethics, and organisational ethics.

### Research ethics

Traditional research ethics in the form of human research ethics committee (HREC) approval or complying with the approved protocol did not often feature in the data. The first approved HREC application as a whole team was celebrated as a milestone, *“… now we’re five researchers.”*-Pat-Academic-FG-18mths. This was also coded under relational ethics. The importance of ensuring that consumer co-researcher roles were clear in the HREC protocol required explanation including clarifying that consumers would not be providing psychosocial support to participants.-Regi-PhD_Candidate-FG-36mths. A new HREC requirement to create what was felt by the team to be an inappropriate volunteer agreement for the consumer co-researchers caused some consternation and was also coded against organisational ethics.-Non-consumer-Log-18mths. When the consumers queried perceived inflexible and time-consuming requirements of the HREC, the importance of complying to avoid delays but then advocating for bigger picture change through a health service committee was discussed.-FN-Mtg19-17mths. This was also coded under organisational ethics.

## Discussion

This qualitative content analysis of an ethnographic study provides a contribution regarding ethical considerations when partnering with consumers in research. It refined Martineau et al.’s existing *Ethical frameworks for patient partnership in research* [2] by examining both consumer and health professional researcher perspectives across a three and half year PhD research partnership. The findings of this study aligned with Martineau et al.’s assertion that partnering with consumers in research is complex and there is a need to move beyond traditional research ethics to include relationships, organisational factors, and research integrity. The refined *Ethical framework for consumer and community engagement in research* retains Martineau et al.’s four original categories [2] but expands on this by depicting the overlapping nature of these concepts when partnering in research. Moreover, the revised framework aims to highlight that relational and organisational ethics were most frequently encountered and require ongoing attention by research teams which are inclusive of consumers. It is recommended that research teams utilise the adaptation of the Martineau et al. (2020) framework presented here to proactively reflect on current and potential ethical issues both individually and as a team.

The notion that ethical considerations are core to CCI and that adopting and promoting a strong ethical ethos is paramount to success [23, 25, 26] was reinforced as the vast majority of qualitative data collected included ethical concepts despite this not being the sole focus of the main ethnographic study [14]. The importance of changing the narrative from ethical issues as a challenge to overcome, to taking a proactive approach that promotes authentic CCI is recommended given how central ethical challenges and opportunities were to the research team interactions, reflections, and evaluations. The following discussion addresses each aspect of the framework in the context of the existing literature and provides recommendations for authentic CCI partnerships with a focus on partnerships between individual consumers and health professional researchers in a research team.

Relational ethics were demonstrated to be a primary consideration for CCI in research as these concepts occurred most frequently in the data. The updated framework highlights relational ethics as the largest circle in the diagram, thereby signposting to research teams that this requires consistent attention. The original Martineau et al. diagram [2] included equal emphasis on each of the four frameworks which may not adequately highlight the primacy of relational ethics in authentic CCI This centrality of relationships has been emphasised by other authors [1, 24, 39]. An approach that actively encouraged trust, consumer perspectives, shared power, and embraced disagreements was beneficial. When the focus was on building meaningful relationships and authentic partnerships, challenges such as the additional time required for meetings and learning were reframed. A virtuous cycle of investing in relationship building and learning led to enjoyment and capability development, and began to influence organisational research culture. Further research focussing on bespoke and ongoing learning for CCI is needed [14, 40]. Patient-oriented research competencies [41], and a capability development framework for successful partnerships in quality improvement [31, 32] may guide learning and development programs but require further investigation.

Emotional reactions to differing perspectives were evident during the study as was the importance of self-awareness and dialogue when these situations occurred in order to support collegial and authentic relationships. Consumer and health professional researcher self-reflection and team reflexivity have been recommended as core to ongoing research team development [1, 11, 25] but seldom formally investigated. This requires attention. An exception was a study where systematic reflective processes were found to enhance consumer leadership for a mental health service evaluation [42]. The impacts of consumer leadership were not evident in the present study. Hence, formal supervision of PhD students by consumers in academic or honorary roles warrants exploration as recommended by others [11, 43].

Absent or unclear organisational policies led to confusion, frustration, and time-consuming negotiations, especially regarding consumer remuneration and HREC processes. Additionally, a lack of resourcing, reward, and recognition of the additional time it takes for authentic CCI was found. Hence organisational ethics is reinforced in the updated framework as a core area for research team focus. The central role of organisations in facilitating positive change in CCI [3, 24, 43] was reinforced by this study as was the concept of ‘ethical distress’ where organisational barriers limited power to act in accordance with research team members’ ethical values [44]. Organisational support including policies, procedures, funding, learning and development initiatives, and reward and recognition of staff and consumers who exemplify meaningful and authentic CCI is critical [25, 45, 46].

Remuneration of consumers for CCI is generally regarded as best practice [11, 47] but was limited in this study. Future research should examine the value-add consumer remuneration brings for all stakeholders, and any unintended ethical challenges or consequences. Evaluation of varied approaches to consumer remuneration such as direct payment, gift cards, and credits for services, is needed due to conflicting opinions and limited research in the area [3, 47]. Budgeting for staff costs for CCI is critical [48]. This warrants further research as this study highlighted the additional time and pressure on health professional researchers resulting from genuine and deep CCI.

The two consumer co-researchers were true partners and there was lived experience within the team across areas such as older age, disability, and healthcare usage. However, diversity regarding socio-economic, educational, and cultural background was highlighted as an ongoing organisational and relational ethics challenge. This was partially addressed through a research advisory group and a health service committee involvement. The dilemma of collaborating meaningfully with more diverse consumers whilst balancing the resources required was not resolved by the research team. Investigating success factors for true research partnerships with cognitively impaired people, consumers from non-English speaking backgrounds, the disabled, Aboriginal and Torres Strait Islander and other First Nations peoples, children, adolescents, and young adults, and/or those who do not have high levels of health literacy is vital [11, 46, 49]. A recently published co-designed framework provides guidance for CCI through a social justice and health equity lens [50]. It reinforces core values discussed throughout this paper such as trust, self-awareness, empathy, relationship building, education, and communication [50] and warrants further investigation.

Similarly to Martineau et al. [2], the positive contribution of CCI to research integrity was demonstrated in this study including in research design, recruitment, data analysis, co-authorship, and co-presentation of findings. In the updated diagram, the relative frequency of the need to address research integrity in terms of team conversations, reflection, and evaluation is depicted as lower than for relational and organisational ethics as per the study findings. Whilst it was a concern reported in Martineau et al.’s scoping review [2], there was no indication that the consumer co-researchers inappropriately influenced research design to the detriment of outcomes in the current study. An awareness of the consumer motivation to drive timely local improvements through presentations and implementation of research findings, in contrast with the academic imperative of generalisable peer reviewed publication was reported as crucial. These different perspectives have been reported before [2, 24]. The consumer co-researchers were perceived to positively influence knowledge dissemination and translation.

Overlapping roles and conflicts of interest dilemmas occurred for both health professional and consumer researchers as noted in the Martineau et al. paper in the research integrity category [2]. In this current study, health professional researcher concern towards a consumer co-researcher who had health issues resulted in unsolicited advice regarding falls prevention. This could be perceived as paternalism which is an important area for self-reflection as it may reinforce inequitable power dynamics and limit consumer opportunities for CCI in a misguided effort to protect from burden or harm [1, 39]. Privacy and confidentiality of both personal and research information were further research integrity issues which arose and have been discussed in other literature [2, 21, 23]. Again, a proactive approach meant that these situations were discussed as a team and a resolution or preventive action taken. Active utilisation of the adapted framework presented here could raise research team awareness of the need to have these important conversations regarding research integrity.

Research ethics is depicted in the updated *Ethical framework for consumer and community engagement in research* as the smallest overlapping circle. This reflects that research ethics considerations rarely required detailed discussion. This does not mean that research ethics were not an important area but may reflect that this area of ethics is well established.

### Limitations

Positionality including a strong commitment to authentic partnerships and awareness of ethical approaches influenced data analysis. This may mean that findings are not generalisable to all partnered research where team members hold different values and perspectives. Most data came from the authors of this paper which is not unusual when examining insider (emic) perspectives in ethnography. Trustworthiness strategies were discussed in the data analysis and positionality sections. The grey area of health professional researchers’ responses, reflections, and reactions being coded in the organisational ethics category as they were organisational actors, but also in the relational ethics category, as they interacted with the team inclusive of consumers, is acknowledged. A further limitation was that whilst consumers contributed to the monthly reflective logs, interviews and focus groups, the health professional researcher perspective may have dominated as the PhD candidate took the field notes and kept the reflexive diary. This point of view was balanced somewhat by consumer collaboration in data analysis. An improvement would be for the consumers to also complete field notes and reflexive diaries. Practical considerations including the time burden [42] and a potential tendency for consumers to find it difficult to focus on the challenges of engagement [16] need to be considered. Consumer diversity regarding socio-economic, educational, and cultural background as discussed above was a limitation in many consumer partnerships and was the case with this study. The updated ethical framework is based on the frequency with which data were coded under ethical categories, but this may not reflect the relative importance or impact of those categories. It is important to note that this research did not consider non-health settings, collective community engagement, and working with Indigenous communities and hence the findings from this research may not be applicable in those contexts.

## Conclusions

This study included consumer and health professional researcher perspectives to refine an existing ethical framework for CCI in research [2]. The updated framework highlighted the complexities of CCI and focused beyond traditional research ethics to include relationships, organisational factors, and research integrity. This paper demonstrated that the narrative of ethical issues being a challenge to overcome in CCI, needs to change. An emphasis on adopting a proactive approach to ethical considerations in CCI promoted authentic team power sharing, reflection, and active communication.

## Supplementary Information

Supplementary Material 2

Supplementary Material 1

## Data Availability

Due to the nature of the consent obtained, the raw data cannot be shared. Summary data which support the findings of this study are available from the corresponding author upon reasonable request.

## Abbreviations

CCI: Consumer and community involvement
FN: Field notes
I: Interviews
FG: Focus groups
Log: Online reflection log
RD: Reflexive diary
