# Supplementary material for: ‘Making little ethical decisions all the time’: examining an ethical framework for consumer and community involvement in research, a co-produced ethnographic study
Source: BMC Med Ethics. 2025 Dec 22;26:177. doi: 10.1186/s12910-025-01355-6 (PMC12751239; doi:10.1186/s12910-025-01355-6)
Supplement: Supplementary file 1 — Supplementary Material 2 [file 12910_2025_1355_MOESM1_ESM.pdf]

Additional File 2: Interview Guide (indicative questions) – 36 months focus group

*Study Title: E*nriching the process and outcomes of a research higher degree through the engagement of consumers: an ethnographic study

Manuscript Title: 'Making little ethical decisions all the time': examining an ethical framework for consumer and community involvement in research, a co-produced ethnographic study

Welcome and Introductions

Thank you for agreeing to participate in this interview which will help describe and evaluate the consumer engagement strategies used in my (Ruth Cox's) PhD research. The interviews are one part of the data being collected in this research project. The interviews and all the other data such as meeting records, logs, field notes and a diary kept by me will be analysed. This analysis will help the whole research team (academic researchers and you as a consumer co-researcher) to check our progress and work out if there are any barriers or ethical issues which we need to address. It will also help us to work out what is going well and what we can build on.

This interview is being recorded for transcription purposes and I will also take notes to support the analysis of the information you provide.

As described in your original Participant Information Sheet, and emphasised through-out this project, I will try to keep what you say anonymous. All responses you give will be transcribed into a written document and you will be given a pseudonym. Given there are only six people in the research team and we know each other well, you need to be aware that people in the research team may be able to identify you even with these measures in place. If you do not wish to answer any of the questions then please let me know. Your participation in this interview will not affect your relationship or position with Metro South Health or Griffith University.

Please be reminded that at any time throughout this interview and the whole research project, you have the right to withdraw your participation without explanation or penalty. It may have been a long time since you signed the study consent form so I would like take this opportunity to make sure that you still consent to participate in this study.

Are there any questions before we begin?

1. **Please tell me what has been happening in the research and with the research team since the last interview / since you joined the research.**

   Example prompts:
   What has been the main thing or things you/the group have been working on?
   How is the group going? How are the group working together?
   What about this group is similar to other groups you have worked with?
   What is different about this group than other groups you have worked with?
   What makes this group different? Eg particular things the groups does, says, behaviours, relationships, interactions?
   Has anything unexpected happened?

- You've now had your last formal meeting as a group and from now on you will meet as needed, rather than regularly, what are your thoughts about that?

- How do you feel about our PhD collaboration coming to an end?

2. **What has worked particularly well and what has helped the research team work well together?**

- What has changed in our group from when we first started working together to now? Think particularly about the last six months.

3. **What hasn't worked so well with the research team and what have been barriers to working well together? Think particularly about the last six months.**

4. **What suggestions for improvement do you have?**

- What would be some advice you would give to a new PhD student and their supervisors about starting this journey?
- What advice would you give to the consumers?

5. **Tell me about the training and support you received as part of this research and what have you learned?**

   Example prompts:
   Who has provided the training and support?
   How has the training and support been provided?
   What has been the content of training or support?
   Has it met your needs?
   Has it met the needs of the research team?
   Can you suggest improvements?

   - Have your learning and support needs changed over time?

6. **What would you like to learn next?**

7. **In what way has the involvement of consumers made a difference to the way the research has been conducted, or to what has happened in or been produced by the project?**

   - Please give specific examples of where you feel the consumer engagement has influenced the research.
     Example prompts:
     Has this had a positive or negative influence or impact on the research?

   - Please give examples of when the consumer engagement has not been able to influence the research, especially given this is a PhD.
     Example prompt:
     Has this had a positive or negative influence or impact on the research?

8. **Thinking about you as an individual, what have been the benefits of your engagement in the research to you personally? Has this changed over time? If so, in what ways?**

    Example prompts:
    What have you have enjoyed?
    What have you have learned?
    Social benefits
    Economic benefits
    Psychological benefits

- What consumer engagement in research activities are you moving on to next now that this research partnership is ending? How has this partnership prepared or not prepared you for that?

9. **Thinking about you as an individual, what have been the challenges of engagement in the research to you personally? Has this changed over time? If so, in what ways?**

    Example prompts:
    What have you have not enjoyed?
    What have you have learned?
    Social challenges
    Economic challenges
    Psychological challenges eg stress, confusion
    Time, other responsibilities

10. **What ethical issues regarding consumers being involved in research have you noticed that need to be considered?**

    Example prompts:
    Are you and the consumers fully informed?
    Did you know what was going on?
    Do you feel free to say no to some things, and/or to say that you no longer want to take part?
    Are there issues with expenses borne by you or the consumers?
    Is the project causing you or anyone else involved any stress or distress?
    Is there enough support available?

- Are there any particular ethical issues to consider given the partnership is coming to an end?

11. **Is there anything else you'd like to say that you haven't had the opportunity to discuss?**
