# Supplementary material for: ‘Making little ethical decisions all the time’: examining an ethical framework for consumer and community involvement in research, a co-produced ethnographic study
Source: BMC Med Ethics. 2025 Dec 22;26:177. doi: 10.1186/s12910-025-01355-6 (PMC12751239; doi:10.1186/s12910-025-01355-6)
Supplement: Supplementary file 2 — Supplementary Material 1 [file 12910_2025_1355_MOESM2_ESM.pdf]

Additional File 1 - Supplementary Table 1: Consumer and community involvement strategies used in each PhD study

Study title	Consumer and community involvement (CCI) details using International Association for Public Participation (IAP2) Spectrum[1] terminology		
	**Preparation**	**Execution**	**Dissemination***
**Research stream 1: capabilities for successful QI partnerships**			
A co-produced capability framework for successful patient and staff partnerships in healthcare quality improvement: results of a scoping review	Two consumers collaborated - refining topic, development of aims	Two consumers collaborated - developing search terms, inclusion criteria, screening one quarter of papers<br><br>Three consumers collaborated - data extraction<br><br>Two consumers collaborated - data analysis, interpretation, planning knowledge translation	Two consumers collaborated - co-authored publication, two consumer organisation newsletters<br><br>Two consumers collaborated – oral co-presentation international conference<br><br>Two consumers collaborated - oral co-presentation two local consumer fora
Refining a capability development framework for building successful consumer and staff partnerships in healthcare quality improvement: a co-produced eDelphi study	Two consumers collaborated - refining topic, development of methods, protocol, ethics submission, recruitment of Research Advisory Group<br><br>One additional consumer consulted - study information sheets, consent forms	Two consumers collaborated - data analysis, interpretation<br><br>Research Advisory Group (three additional consumers) involved in - refining recruitment strategies, data collection tools, interpretation findings, planning knowledge translation	Two consumers collaborated - co-authored publication and two consumer organisation newsletters<br><br>Two consumers collaborated - co-presented to local staff-consumer partnership committee

Study title	Consumer and community involvement (CCI) details using International Association for Public Participation (IAP2) Spectrum[1] terminology		
	**Preparation**	**Execution**	**Dissemination***
Learning and development needs for successful staff and consumer partnerships on healthcare quality improvement committees: a co-produced cross-sectional online survey	Two consumers collaborated - refining topic, development of methods, protocol, ethics submission<br>Three additional consumers on staff-consumer partnership committee consulted – methods development	Two consumers collaborated - data analysis, interpretation, planning knowledge translation<br>Three consumers on staff-consumer partnership committee consulted – data interpretation, planning knowledge translation	Two consumers collaborated - co-presented to two local staff-consumer partnership committees<br>Two consumers collaborated – co-authored publication, oral co-presentations international conference, two national conferences, two local symposia
**Research stream 2: consumer engagement in Research with a focus on PhDs**			
Consumer engagement in occupational therapy health-related research: A scoping review of the Australian Occupational Therapy Journal and a call to action	-	Two consumers collaborated - finalising data analysis, leading theme naming, interpretation, planning knowledge translation	Two consumers collaborated - co-authored publication, two consumer organisation newsletters<br>Two consumers collaborated - oral co-presentation two international oral and one national conference poster
Different in so many ways: exploring consumer, health service staff, and academic partnerships in a Research Advisory Group through rapid ethnography	Two consumers collaborated - refining topic, development of methods, protocol, ethics submission, recruitment of Research Advisory Group	Two consumers collaborated - data analysis, interpretation, planning knowledge translation	Two consumers collaborated - co-authored publication<br>Two consumers collaborated - oral co-presentation two local health service workshops, one university staff learning session

**Study title**	**Consumer and community involvement (CCI) details using International Association for Public Participation (IAP2) Spectrum[1] terminology**		
	**Preparation**	**Execution**	**Dissemination***
	One additional consumer consulted - study information sheets, consent form		
Enriching the process and outcomes of a research higher degree through the engagement of consumers: an ethnographic study	Four consumers consulted - topic, methods<br><br>Three consumers consulted - recruitment procedures, study information sheet, consent form	Three consumers collaborated - refining data collection tools, procedures<br><br>Two consumers collaborated - data analysis, interpretation, planning knowledge translation	Two consumers collaborated - co-authored one publication, two consumer organisation newsletters<br><br>Two consumers collaborated - oral co-presentation two international and one national conference; three local health service workshops; two university staff seminars; one national webinar<br><br>Two consumers collaborated - oral co-presentation three local symposia<br><br>Two consumers involved – local videoed learning resource

*Many social media posts were additional to these dissemination activities

1. International Association for Public Participation Australasia. IAP2's public participation spectrum Toowong, Queensland: International Association for Public Participation Australasia; 2018 [Available from: https://iap2.org.au/wp-content/uploads/2020/01/2018_IAP2_Spectrum.pdf]
